# Metformin at the Time of COVID‐19 Infection and Risk of Long COVID: A Target Trial Emulation Study

**DOI:** 10.1002/jmv.71190

**Published:** 2026-10-29

**Authors:** Carolyn T. Bramante, Til Stürmer, Jared D. Huling, John B. Buse, Christopher Lindsell, Thomas Stewart, Russell L. Rothman, David Sahner, Sarah E. Dunsmore, Eric Topol, Talia D. Wiggen, Steve Makkar, Andrew Toler, Taylor Estepp, Steven G. Johnson, N3C Consortium

**Affiliations:** ^1^ Department of Medicine Division of General Internal Medicine, University of MN Medical School Minneapolis Minnesota USA; ^2^ Department of Epidemiology, Gillings School of Global Public Health University of North Carolina at Chapel Hill Chapel Hill North Carolina USA; ^3^ Division of Biostatistics and Health Data Science University of Minnesota School of Public Health Minneapolis Minnesota USA; ^4^ Department of Medicine, Division of Endocrinology University of North Carolina School of Medicine Chapel Hill North Carolina USA; ^5^ Division of Biostatistics and Bioinformatics Duke University School of Medicine Durham North Carolina USA; ^6^ Division of Biostatistics University of Virginia School of Data Science Charlottesville Virginia USA; ^7^ Vanderbilt Institute for Medicine and Public Health Nashville Tennessee USA; ^8^ National Center for Advancing Translational Science (NCATS) Bethesda Maryland USA; ^9^ NCATS contractor Axle Informatics Rockville Maryland USA; ^10^ Scripps Research Translational Institute La Jolla California USA; ^11^ Institute for Health Informatics, University of Minnesota Minneapolis Minnesota USA

## Abstract

Our objective was to evaluate metformin prescribed at the time of SARS‐CoV‐2 infection on the risk of developing Long COVID (LC) in electronic health record data. We conducted a new user analysis of metformin prescribed within 6 days of documented infection with severe acute respiratory syndrome coronavirus 2 (SARS‐CoV‐2) versus experimental control: prescription for fluvoxamine, fluticasone, ivermectin, or montelukast. Inclusion criteria: a clinic visit in the 0–6 months and the 6–12 months before infection. Exclusion criteria: metformin or control within 12 months. Primary outcome: LC or death (LC/D), to address death as a competing risk, among patients prescribed drug within Days 0–6 of infection. LC was defined by diagnosis code or computable phenotype. We used entropy balancing to estimate the average treatment effect with a weighted log‐linear model. After weighting, there were 248 in the metformin and control groups; the average age was 53 (16); 16% were Black; and 16% were Hispanic. In the primary analysis, 10/248 (4.0%) in the metformin group developed LC/D versus 21/248 (8.5%) in the control group, adjusted risk ratio (aRR) 0.47 (95% CI 0.25–0.89, *p* = 0.02). For prescriptions on Days 0–1 relative to infection, aRR was 0.39 (95% CI 0.12–1.24); for prescriptions on Days 0–14, the aRR was 0.75 (95% CI 0.52–1.08). In this observational analysis, metformin prescribed within a week of documented SARS‐CoV‐2 infection was associated with a 53% lower risk of LC over 6 months than comparator medications. With the aRR of 0.47 (95% CI 0.25–0.89), any risk reduction between 75% and 11% is highly compatible with our data. This analysis of electronic health record diagnoses is important for the reproducibility of clinical trial results that ascertained the same outcome but via participant‐report.

## 1 Background

The severe acute respiratory syndrome coronavirus 2 (SARS‐CoV‐2) continues to infect adults and children worldwide [1, 2, 3]. Long COVID (LC) symptoms often include functional limitations and impairment [1, 4, 5, 6, 7, 8]. Even mild limitations after SARS‐CoV‐2 infection could have broad societal and environmental impacts [4, 6, 9]. LC may be a greater predictor than severe acute COVID‐19 for having post‐COVID economic hardship [10]. The potential for LC to increase economic disparities highlights the urgent need to understand whether medications at the time of SARS‐CoV‐2 infection affect the risk of developing LC.

Metformin has inhibited SARS‐CoV‐2 in several in vitro studies [11, 12, 13]. In a randomized clinical trial (RCT) evaluating metformin versus placebo in over 1000 outpatient adults (COVID‐OUT), LC occurred in 6.2% of metformin versus 10.3% of placebo subjects by Day 300 [14, 15]; and 14% of metformin versus 23% of the placebo group had detectable nasal viral load on Day 10 [16]. However, only 22% of the trial participants were enrolled during the Omicron era, enrollment was restricted by age (30–85 years) and body mass index (BMI) ≥ 25 kg/m^2^, and the LC outcome was diagnosis by a clinician. Thus, our objective was to use electronic health record (EHR) data to understand the impact of new metformin use at the time of SARS‐CoV‐2 infection on the risk of developing clinician‐diagnosed LC in a broader sample to improve generalizability beyond patients who consent to being in a clinical trial. While randomization removes confounding from the comparison of intervention versus placebo in a clinical trial, the comparison is still limited by the selection that is inherent to clinical trials. EHR data are uniquely suited for reproducing clinical trial findings when the outcomes are clinical outcomes that are captured in the EHR.

When using EHR data, it may not be possible to determine the exact reason a medication was prescribed, and thus whether the medication was taken during the timeframe of interest (i.e., during acute infection).

To this end, we restricted the analysis to patients without an indication for chronic metformin use who were newly prescribed this medication at the time of acute COVID‐19. When considering why metformin might be prescribed at the time of SARS‐CoV‐2 infection before the RCT results, several mechanistic papers on metformin and COVID‐19 were published early in 2020, including a February 2020 letter in *Lancet* suggesting metformin as a safe host‐directed immunomodulator [17, 18, 19, 20, 21]. Observational analyses of prevalent metformin use and associations with less severe outcomes during acute COVID‐19 were published in early 2020 [18, 22, 23, 24]. The Comprehensive Diabetes Center at the University of Alabama at Birmingham published a manuscript in July 2020 reporting an association between metformin and lower mortality from COVID‐19 (OR 0.33; 95% CI 0.13–0.84; *p* = 0.0210) [25]. These manuscripts may have led to prescriptions for metformin for acute infection.

Previous observational analyses that assessed LC as an outcome have not used an active comparator, or “experimental control” [26, 27, 28]. An experimental control reduces unmeasured confounding, such as likelihood of engagement with healthcare, immortal time bias, and the placebo effect, more effectively than an untreated control [29, 30, 31]. The placebo effect is influential on both subjective and objective laboratory outcomes [32, 33]. If an experimental control is not used, the placebo effect is only experienced in the exposure group and not the control group, biasing away from the null. We emulated a placebo‐controlled trial by analyzing new use of metformin versus new use of an experimental control.

## 2 Methods

### 2.1 Design

Target trial emulation (TTE) in adults with documented SARS‐CoV‐2 infection in the National COVID Cohort Collaborative (N3C) database. The N3C is a centralized and harmonized database of de‐identified EHR data from over 21 million individuals from every state in the United States (details in Supplement S1) [34, 35]. The target trials emulated are the COVID‐OUT and ACTIV‐6 trials, both randomized, blinded, decentralized trials of metformin versus exact‐matching placebo to assess whether metformin during acute infection prevents LC [14, 15].

TTE is a methodological approach for the design of observational studies that aims to align the structure and intent of the observational study to that of the idealized RCT, minimizing discrepancies in their findings by ensuring they aim to answer the same question [36]. By explicitly specifying eligibility criteria, treatment initiation, follow‐up, outcome assessment, and target estimand as an RCT would, TTE can reduce common biases and pitfalls in observational studies such as immortal time bias and confounding [37]. This alignment improves the validity of real‐world evidence and enhances the comparability between observational and trial results, fostering more reliable insights into treatment effects [38].

The follow‐up in this TTE closely emulates the target trials, in which participants did not receive therapy at the outset of symptoms or immediately upon a positive test. In those decentralized trials, the follow‐up period started on the day the study drug was delivered, so participants who were hospitalized between consent and receiving drug in the mail were not eligible (Table 1 and Figure 1). The index dates for inclusion in the TTE ranged from March 2020 to September 2023. The date of the first documented SARS‐CoV‐2 infection, as defined by the N3C (positive PCR or COVID‐19 diagnosis code) [34], is the index date [39]. Day 1 of follow‐up was the prescription of metformin or control.

**Table 1:** Individuals who experienced death before the prescription could be started were excluded from the trial emulation to mimic both target trials (COVID‐OUT and ACTIV‐6), as they were both decentralized trials that entailed medication delivery to the home.

Approaches	Target trial ACTIV‐6 (10/12/23– 4/29/24)	Target trial COVID‐OUT (12/31/21–1/28/22)	Target trial emulation (03/01/2020–09/30/2023)
Eligibility criteria	Documented SARS‐CoV‐2 Enrolled within 10 days of documented infection Enrolled within 7 days of symptoms Age 30–85 No current metformin use	Documented SARS‐CoV‐2 Enrolled within 3 days of documented infection Symptoms not required, but if present, < 7 days Age 30–85 No current metformin use Body mass index < 25 kg/m^2^ was an exclusion Known prior COVID‐19 infection was an exclusion	Documented SARS‐CoV‐2 Prescription within 6 days (primary analysis population), secondary analyses with prescriptions within 1 day and 14 days Age > 18 years No metformin or comparator prescription within 12 months No minimum body mass index The infection analyzed is the first documented infection
Intervention	Metformin immediate release or exact‐matching placebo tablets	Metformin immediate release or exact‐matching placebo tablets	Any metformin prescription or active comparator (medications with no convincing evidence of favorable impact on acute COVID‐19 outcomes: fluvoxamine, ivermectin, fluticasone, montelukast)
Treatment assignment	Randomization	Randomization	Prescription for metformin or comparator placed; propensity scores to balance measured covariates across treatment cohorts
Considerations for medication acquisition	Delivery of medication takes 2–3 days on average	Delivery of medication takes 1 day on average	Getting a medication prescription then obtaining it from a pharmacy likely takes 0–3 days on average
Post‐randomization exclusions	Hospitalized at the time of medication delivery Delivery failure Death before Day 1	Hospitalized at the time of medication delivery Delivery failure Death before Day 1	Hospitalized between −3 days and +1 days of infection because this would preclude the ability to fill a prescription Death on or before Day 1
Day 1	First day of a medication dose	First day of a medication dose	Day prescription placed
Follow‐up	Days 1–180	Days 1–300	Days 1–180

*Note:* Those who experienced hospitalization between −3 and 1 days were also excluded to mimic the target trial post‐randomization exclusions that were necessary because the decentralized design created time between randomization and the intervention in the RCTs.

**Figure 1:**
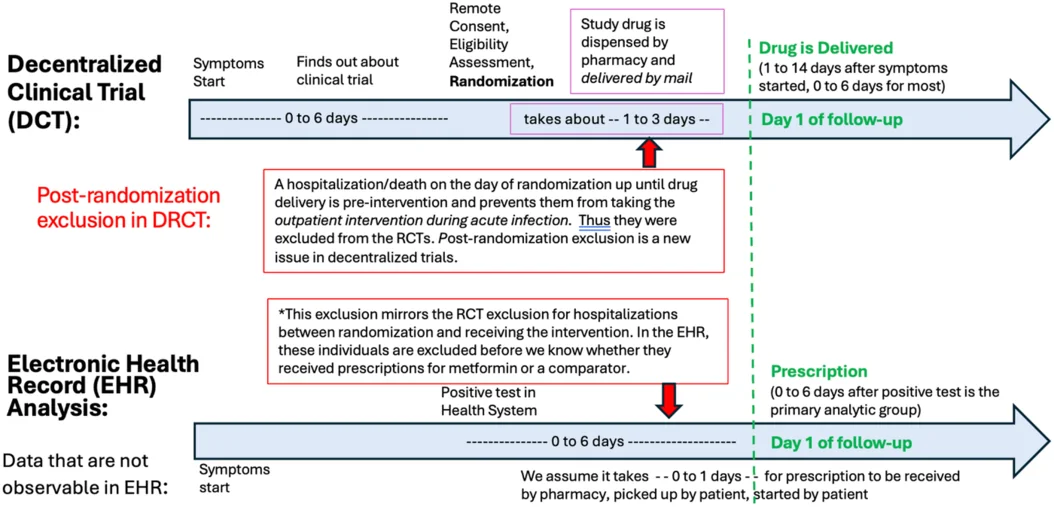
This is a visual overview of practical constraints that exist when conducting a decentralized clinical trial (DCT) and an electronic health record (EHR) analysis. Because eligibility assessments, consent, randomization, and starting study drug do not happen at the same time in DCTs, there may be post‐randomization exclusions.

### 2.2 Experimental Control and Intention to Treat

We emulated placebo control with medications that have been used for acute SARS‐CoV‐2 infection but do not have consistent clinical trial data to support an effect on preventing Long COVID‐19: fluvoxamine, ivermectin, montelukast, or fluticasone (inhaled and intranasal) [15, 40, 41]. The primary exposure of interest was a new metformin prescription on Days 0–6 of documented SARS‐CoV‐2 infection. The experimental control was new prescription of one of the control medications on Days 0–6 of documented infection. Individuals subsequently prescribed metformin or a control remained in the cohort of their first prescription, in keeping with intention to treat in a clinical trial. Individuals prescribed multiple control medications were not treated differently and were categorized according to their first control prescription.

### 2.3 Analysis Population

Documented SARS‐CoV‐2 infection as defined in the N3C [34], and evidence of two outpatient visits prior to the index date: one in the 0–6 months prior and one in the 6–12 months prior, to increase the likelihood that comorbidities and pre‐existing prescriptions were captured. Per new‐user methodology, those with a prescription for metformin or a control in the 12 months prior to the index were excluded [36]. To mimic prospectively starting an intervention, we excluded patients with a documented clinical indication for metformin to minimize the potential of prevalent metformin use in the comparator group, and to enrich the sample for individuals starting the intervention at the time of the indication of interest (SARS‐CoV‐2 infection).

Metformin is the first‐line pharmacologic agent for type 2 diabetes and prediabetes. Diabetes and diabetes treatments are strongly associated with acute COVID‐19 severity and LC, so excluding those with diabetes from both the exposure and control groups is important because measures to address confounding would be insufficient to address the imbalance between metformin and experimental controls [42, 43]. We also excluded polycystic ovary syndrome, antipsychotic induced weight gain, and gestational diabetes (other indications for which metformin is often prescribed).

Patients with indications for the controls were not excluded because no prospective data suggest that the controls prevent the development of LC, and the control agents are not first‐line treatments for their most common indications [44, 45]. Individuals prescribed a control at the time of documented infection are more likely to have been prescribed and thus taken the medication for COVID‐19 rather than a chronic indication. The most common indications for the control agents (asthma and allergic rhinitis) include symptoms common during acute COVID‐19. Therefore, if an individual were coincidentally prescribed fluticasone for allergic rhinitis at the time of SARS‐CoV‐2 infection, they might obtain and start that prescription right away. In contrast, patients with type 2 diabetes and prediabetes do not frequently have symptoms that are common during acute COVID‐19 and therefore may be less likely to start metformin for a chronic indication during acute illness [46]. Key to emulating a clinical trial is finding individuals who initiate the treatment of interest during the timeframe for the indication of interest (acute infection) [36].

Contraindications for metformin or controls were excluded (Stage 4 or 5 chronic kidney disease, Figure 2) [47]. Data from sites which had never reported an *International Statistical Classification of Diseases Tenth Revision* (*ICD‐10*) code U09.9 for post‐COVID‐19 condition were excluded to minimize ascertainment error. Individuals who died or developed LC before treatment were excluded, as were individuals with a hospitalization from Days −3 to 1 relative to the positive SARS‐CoV‐2 test because they would be unlikely to have started metformin or an experimental control. This emulates the COVID‐OUT and ACTIV‐6 trials, as both decentralized trials excluded those hospitalized at the time study drug was delivered [15, 48].

**Figure 2:**
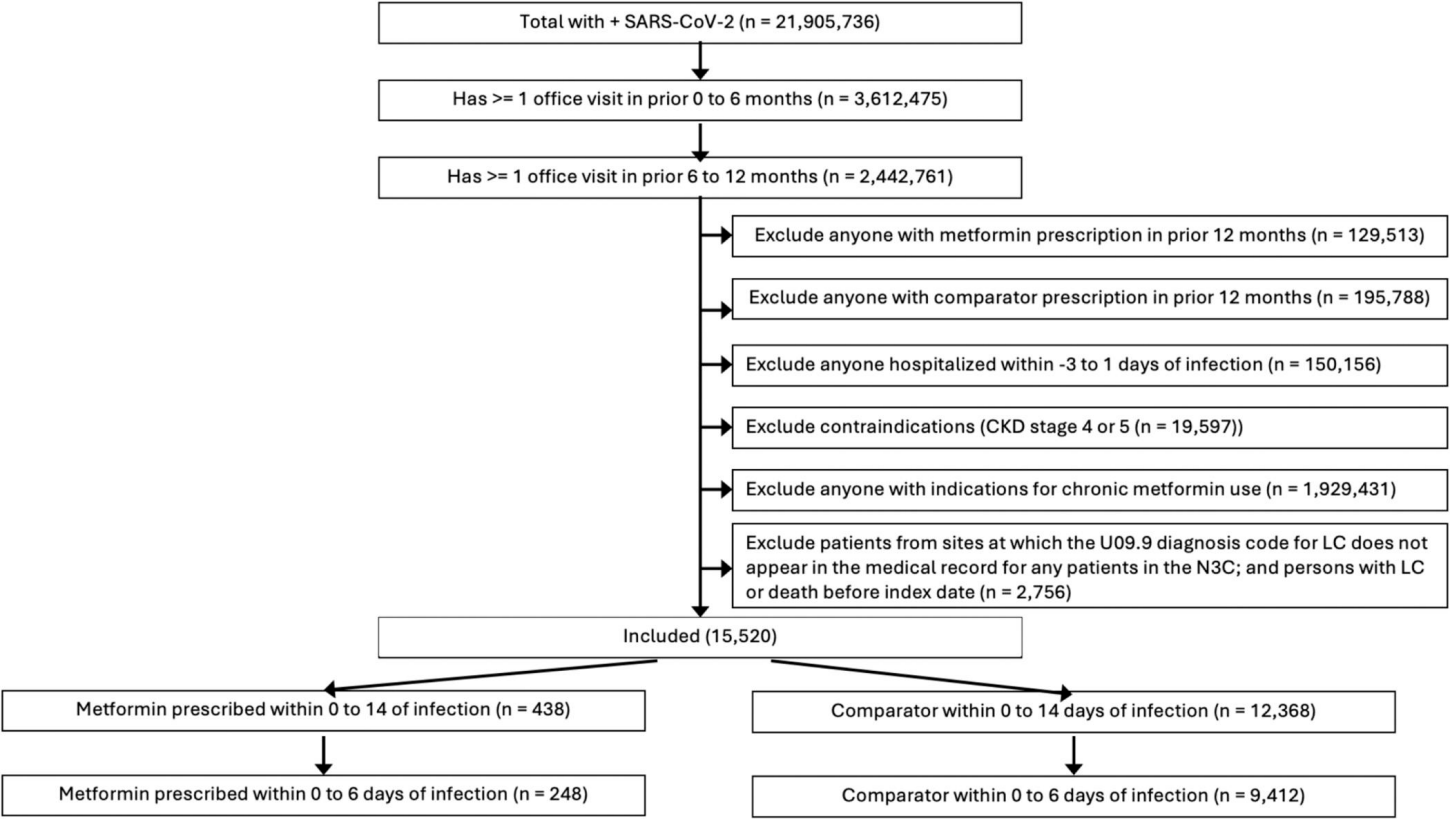
Cohort attrition for the metformin and active comparator (control) cohorts. Days 0–6 were chosen a priori as the window that likely most closely matches most participants in the target trials, the lack of symptom data in the medical record makes it impossible to match exactly. The number who died before the treatment window was too small to show while maintaining N3C data privacy rules, so that number is included with the number excluded from sites that have never reported a U09.9 code (those sites are excluded because of potential misclassification of the outcome).

### 2.4 Outcome

Incidence of LC or death (LC/D) within 180 days of the metformin or control prescription. Because the endpoint is death or LC, death is not a competing risk. LC was defined as an ICD‐10 diagnosis code for LC (U09.9) or a computable phenotype for LC that is based on symptoms and conditions [34, 49, 50]. The computable phenotype is based on new diagnoses, such as post‐viral fatigue or new‐onset asthma; or worsening of preinfection diagnoses. The worsening is calculated by looking at equal windows of time before and after infection and the number of clinic visits for that condition in the preinfectious window compared to the postinfectious window. In an observational study of prevalent metformin use in adults with type 2 diabetes, the overall incidence of this computable phenotype was 4.9% and of U09.9 was 1.7%; the relative risk reduction with prevalent metformin exposure was similar for U09.9, hazard rato (HR) 0.79, versus HR 0.85 for the computable phenotype [51].

### 2.5 Statistical Analysis

To balance covariates and adjust for confounding between cohorts, we used the R weightit package (version 0.14.2) to perform entropy balancing (EB), allowing an estimation of the average treatment effect among the treated (ATT) [52, 53, 54] using a weighted log‐linear model. EB works similarly to inverse propensity score weighting: weights are directly estimated by minimizing a measure of entropy divergence of the control weights relative to base (e.g., uniform) weights in the population, subject to exact balance of covariate distribution moments in the treated and control groups. Although EB weights are constructed via an optimization criterion, they implicitly estimate the propensity score [54]. We weighted on demographics (age, gender, race/ethnicity), BMI, prior medications and conditions that have associations with LC, and laboratory values including hemoglobin A1c (Table 2 and Tables S3 and S4).

**Table 2:** Demographic characteristics in those with a prescription on Days 0–6 from infection; *n* (%).

		Before weighting			After weighting		
		Metformin (*n* = 248)	Control (*n* = 9412)	SMD	Metformin (*n* = 248)	Control (*n* = 248.1)	SMD
Age, mean (SD)		53.31 (16.48)	45.28 (20.66)	0.430	53.31 (16.45)	52.74 (17.25)	0.034
Age category	< 21	< 10	1288 (0.14)	0.383	< 10	< 10	0.000
	21–45	< 70	3454 (0.37)	0.200	68 (0.27)	68 (0.27)	0.000
	46–65	116 (0.47)	2964 (0.31)	0.317	116 (0.47)	116 (0.47)	0.000
	≥ 66 years	56 (0.23)	1702 (0.18)	0.112	56 (0.23)	56 (0.23)	0.000
Female		113 (0.46)	6310 (0.67)	0.444	113 (0.46)	113 (0.46)	0.000
American Indian or Alaska Native		< 10	42 (0.00)	0.007	< 10	< 10	0.000
Asian		< 10	236 (0.03)	0.063	< 10	< 10	0.000
Black or African American		40 (0.16)	1638 (0.17)	0.034	40 (0.16)	40 (0.16)	0.000
Native Hawaiian, or Pacific Islander		0 (0.00)	24 (0.00)	0.072	0 (0.00)	< 10	0.009
Hispanic or Latino Any Race		39 (0.16)	1251 (0.13)	0.069	39 (0.16)	39 (0.16)	0.000
White non‐Hispanic		153 (0.62)	5807 (0.62)	0	153 (0.62)	153 (0.62)	0.000
*Number of office visits before infection, mean (SD)*							
0–6 months before infection		6.28 (9.56)	7.87 (9.89)	0.163	6.28 (9.54)	7.38 (10.94)	0.107
6–12 months before infection		5.19 (7.95)	7.34 (9.17)	0.251	5.19 (7.94)	6.38 (8.52)	0.144
Body mass index (BMI) n (%)	BMI, mean (SD)	35.91 (9.30)	29.64 (8.26)	0.712	35.91 (9.27)	35.90 (11.05)	0.000
	18.5–24.9	< 10	1651 (0.18)	0.483	< 10	14 (0.06)	0.120
	25.0–29.9	< 29	1849 (0.20)	0.233	< 29	29 (0.12)	0.007
	≥ 30.0	94 (0.38)	2907 (0.31)	0.148	94 (0.38)	88 (0.35)	0.055
	Missing	117 (0.47)	2654 (0.28)	0.399	117 (0.47)	117 (0.47)	0.000
*Medications prior to infection* a							
Serotonin reuptake inhibitors		18 (0.07)	1197 (0.13)	0.183	18 (0.07)	18 (0.07)	0.000
Angiotensin converting enzyme inhibitors		21 (0.08)	592 (0.06)	0.083	21 (0.08)	21 (0.08)	0.000
Angiotensin receptor blockers		18 (0.07)	552 (0.06)	0.056	18 (0.07)	18 (0.07)	0.000
Statins		18 (0.07)	1065 (0.11)	0.140	18 (0.07)	18 (0.07)	0.000
Anticoagulant		14 (0.06)	492 (0.05)	0.018	14 (0.06)	14 (0.06)	0.000
Aspirin		11 (0.04)	414 (0.04)	0.002	11 (0.04)	11 (0.04)	0.000
Steroids		61 (0.25)	2776 (0.29)	0.110	61 (0.25)	61 (0.25)	0.000
GLP‐1 RA		< 10	44 (0.01)	0.140	< 10	< 10	0.000
Outpatient insulin		14 (0.06)	73 (0.01)	0.279	14 (0.06)	14 (0.06)	0.000
SGLT‐2 inhibitor		< 10	12 (0.00)	0.054	< 10	< 10	0.001
*Clinical conditions prior to infection*							
Coronary artery disease		15 (0.06)	564 (0.06)	0.002	15 (0.06)	15 (0.06)	0.000
Cancer		12 (0.05)	660 (0.07)	0.092	12 (0.05)	12 (0.05)	0.000
Chronic kidney disease, Stages 1–3		< 10	341 (0.04)	0.126	< 10	< 10	0.000
Heart failure		10 (0.04)	342 (0.04)	0.021	10 (0.04)	10 (0.04)	0.000
Hypertension		71 (0.29)	2850 (0.30)	0.036	71 (0.29)	71 (0.29)	0.000
Metabolic liver disease		10 (0.04)	315 (0.03)	0.036	< 10	< 10	0.000
Insomnia		< 10	308 (0.03)	0.003	< 10	< 10	0.000
Depression		21 (0.08)	1295 (0.14)	0.169	21 (0.08)	21 (0.08)	0.000
Asthma		23 (0.09)	1970 (0.21)	0.330	23 (0.09)	23 (0.09)	0.000
COPD		< 10	511 (0.05)	0.087	< 10	< 10	0.000
Last hemoglobin A1c, mean (SD)		7.09 (2.39)	5.39 (0.52)	0.983	7.09 (2.37)	7.09 (2.76)	0.000
Creatinine, mean (SD)		0.92 (0.57)	0.93 (0.72)	0.011	0.92 (0.57)	0.92 (0.41)	0.000
*COVID variant epoch (not used for weighting)*							
Ancestral (Mar–Sept 2020)		21 (0.08)	391 (0.04)	0.178	21 (0.08)	21 (0.08)	0.001
Alpha (Oct 2020–May 2021)		70 (0.28)	1651 (0.18)	0.256	70 (0.28)	49 (0.20)	0.203
Delta (June 2021–Nov 2021)		34 (0.14)	1691 (0.18)	0.117	34 (0.14)	49 (0.20)	0.162
Omicron (starting Dec 2021)		123 (0.50)	5664 (0.60)	0.214	123 (0.50)	129 (0.52)	0.051
*Days from infection to treatment (not used for weighting*; > *5 days not shown)*							
Comparator	0	< 10	5751 (0.61)	1.557	< 10	127 (0.51)	1.257
	1–3	< 10	2165 (0.23)	0.613	< 10	67 (0.27)	0.703
	3–5	< 10	1018 (0.11)	0.438	< 10	35 (0.14)	0.521
Metformin	0	94 (0.38)	0 (0.00)	1.105	94 (0.38)	0.0 (0.00)	1.105
	1–3	85 (0.34)	0 (0.00)	1.021	85 (0.34)	0.0 (0.00)	1.021
	3–5	43 (0.17)	< 10	0.647	43 (0.17)	< 10	0.625

Abbreviations: COPD = chronic obstructive pulmonary disease, SMD = standard mean difference, absolute.

^a^ Not shown: other diabetes medications, which were all < 10 before weighting for both cohorts. The full list of all variables used in weighting is in Tables S3 and S4.

Because missingness was high for BMI, and missingness may be a proxy for medicalization and data quality, an indicator for the missingness of BMI was included in the weighting. We also weighted by N3C site to account for site differences in data practices around BMI or other data. We did not add an indicator for missingness for other variables that most EHR records had (age, sex) because missingness was low. Love plots were generated to visualize the balance achieved among cohorts on the covariates (Figure S1). Because fewer than 10% of sites in the N3C have reliable data on vaccination status, this important variable was not included. The R svyglm package (version 4.0) was used to fit a weighted log‐linear model to estimate the risk ratio of LC/D between patients exposed to metformin versus the controls.

Stratified analyses of interest were viral variant epoch and those with new use of nirmatrelvir–ritonavir [55]. A sample‐size calculation was not performed because we had no reason to not analyze all individuals who remained after applying the inclusion and exclusion criteria.

### 2.6 Sensitivity Analyses by Time to Treatment

A time to treatment of 0–6 days from documented infection was prospectively chosen as the primary analytic sample to emulate the exclusion of individuals with ≥ 7 days of symptoms in the target trials. In the COVID‐OUT trial, initiation of study drug within 4 days of symptom onset, as opposed to ≥ 4 days from symptom onset, showed a smaller HR for metformin [14, 15]. To examine whether the duration of time between documented infection and prescription influenced the results in this TTE, we assessed two additional treatment windows (Days 0 to +1 and Days 0 to +14). We did not use mutually exclusive timeframes because of concern for sample size, but the windows would be expected to contain a progressively larger number of patients who initiated therapy later, with the greatest number of “late starters” in the 0–14‐day group. We examined only the Days 0–6 and 0–14 treatment windows within subgroups because the Day 0–1 window subgroup was not large enough to balance.

### 2.7 Sensitivity Analyses by Experimental Controls (Excluding Fluvoxamine; Excluding Ivermectin)

The research team became aware that some individuals seeking ivermectin for COVID infection may be less likely to seek care for LC symptoms or to allow symptoms to be ascribed to LC [15]. Ascertainment bias could lead to misclassification of the outcome, so subgroup analysis excluded ivermectin from the control cohort. Because of emerging data about selective serotonin reuptake inhibitors (SSRIs) [56], we excluded fluvoxamine from the control cohort in a sensitivity analysis as well.

## 3 Results

After applying the inclusion and exclusion criteria, the primary sample was *n* = 248 in the metformin cohort and *n* = 9412 in the control cohort (Figure 2). Missingness was frequent for BMI (47% in the metformin group and 28% in the control group), and few patients in the metformin group had a documented BMI of < 25 kg/m^2^. Before weighting, the metformin group was older, had a higher mean BMI and mean HbA1c; and a larger portion was male (Table 2).

After weighting, these variables were well balanced, and the median standardized mean difference (SMD) was 0 (Table 2). After weighting, 16% of the metformin and control groups were Black; 16% of both groups were Hispanic; the mean BMI in the metformin group and control groups were, respectively, 35.9 (SD 9.27) and 35.9 (SD 11.05); and 50% of the metformin cohort and 52% of the control cohort were infected during the Omicron variant epoch. The modal day of prescription was Day 0 for both metformin (38%) and the controls (61%). Most of the metformin (72%) and control (84%) prescriptions were on Days 0–2 relative to documented infection. A minority of the metformin (10%) and control (5%) prescriptions were on Day 5 or later (Table 2).

The primary analytic sample was those receiving a metformin or comparator prescription within Days 0–6 relative to infection. The unweighted overall incidence of LC/D was 7.0%, with 1.7% deriving from death and 5.5% from LC. The numerators and denominators are not given due to the N3C data governance rules for counts in a cell of a table (at least 10) (Table S2).

The weighted overall incidence of LC/D in the Days 0–6 cohort was 6.3%: 4.0% in the metformin cohort and 8.5% in the experimental control cohort, with absolute risk reduction of 4.5% for metformin versus control (Table S2).

In the primary analysis, the adjusted risk ratio (aRR) for LC/D was 0.47 (95% confidence interval [CI] 0.25–0.89, *p* = 0.02) for metformin versus control. The aRR was 0.37 (95% CI 0.12–1.24) for a time to treatment window of 0–1 days relative to infection; and 0.75 (95% CI 0.52–1.08) for 0–14 days (Figure 3). In the Days 0–6 treatment window, the aRR for the Omicron era was 0.46 (95% CI 0.14–1.50); the aRR for the pre‐Omicron eras combined was 0.41 (95% CI 0.18–0.92). The subgroup of those with a prescription of nirmatrelvir–ritonavir was too small to balance; in the subgroup excluding those with new use of nirmatrelvir–ritonavir, the aRR was 0.48 (95% CI 0.25–0.60). The results were consistent across sensitivity analyses (Figure 3 and Figures S2–S8).

**Figure 3:**
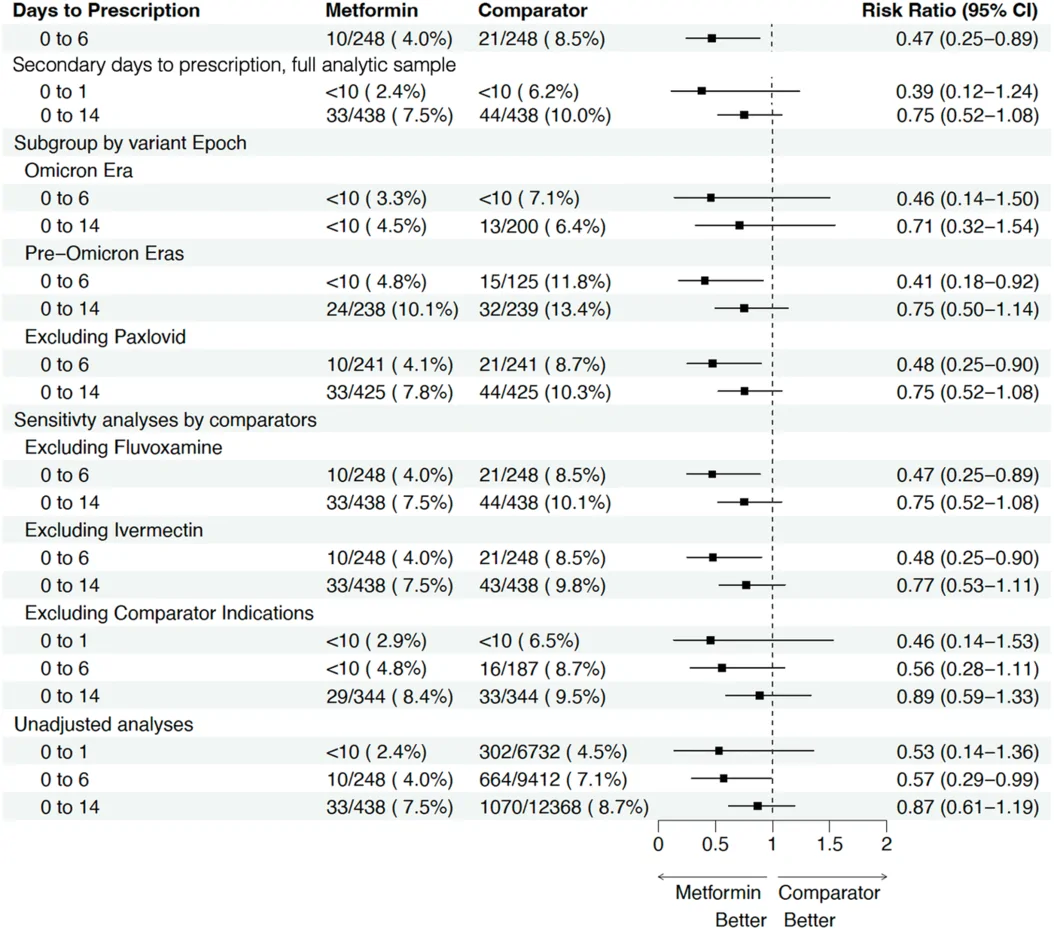
This is a forest plot showing metformin use versus control use and the development of Long COVID/Death by Day 180. The black squares represent risk ratios (RR), and the lines represent 95% confidence intervals. The number of outcomes, denominators, and exact percentages are sometimes omitted so that cell sizes < 10 are not able to be calculated, in accordance with the data use requirements in the National COVID Cohort Collaborative (N3C). The pre‐Omicron Era is larger than the Omicron era in both the metformin and control cohorts, but the denominator for the Days 0–6 sample is not shown because of cell sizes < 10. The subgroup of those with new nirmatrelvir–ritonavir use was too small to analyze, so we present only the subgroup who did not also receive a prescription for nirmatrelvir–ritonavir.

Figure 4 displays the cumulative incidence of LC/D cases. In the primary analytic sample, the HR was 0.48 (95% CI 0.25–0.92). For the sample with prescriptions on Days 0–1 relative to infection, HR was 0.39 (95% CI 0.12–1.26); for prescriptions on Days 0–14, the HR was 0.75 (95% CI 0.51–1.10).

**Figure 4:**
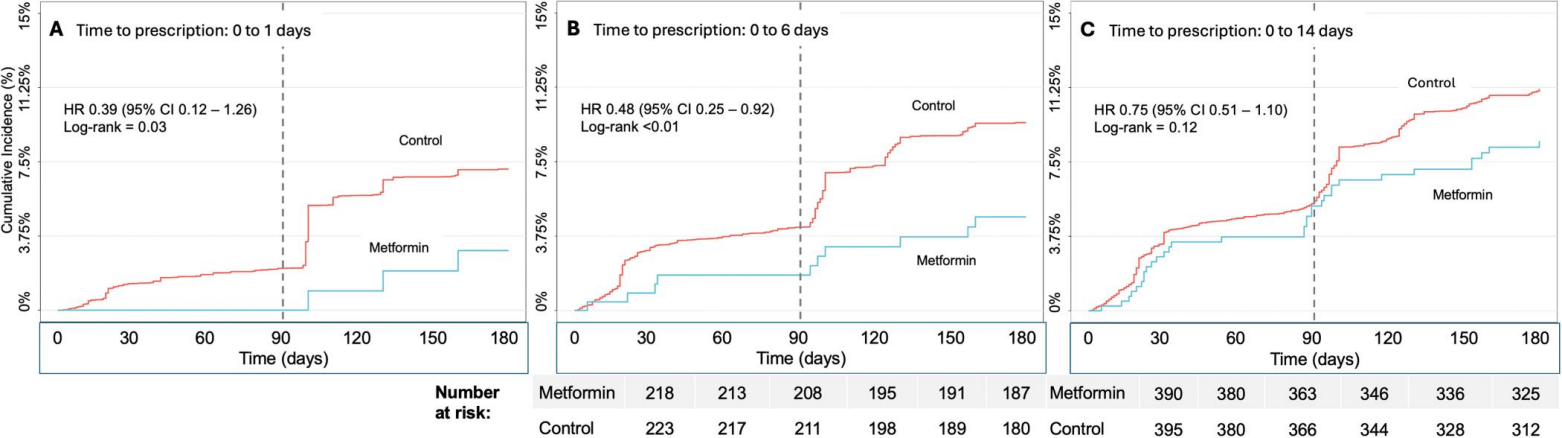
Cumulative incidence curves for the outcome of Long COVID/Death in the 180 days after a metformin prescription (blue) or control prescription (red). (A) is the cohort who received a prescription on Days 0–1 from infection; (B) is 0–6 from infection; and (C) is 0–14 from infection. The increase in outcomes at Day 90 reflects the computable phenotype, which cannot be computed until 90 days after infection. Per data use requirements in the National COVID Cohort Collaborative (N3C), the number at risk is not shown for the 0–1 cohort, nor for the starting cohort sizes for the 0–6 and 0–14, due to small cell sizes or the ability to calculate small cell sizes.

## 4 Discussion

We conducted a target trial analysis of new metformin use versus an experimental control at the time of SARS‐CoV‐2 infection to emulate the COVID‐OUT and ACTIV‐6 RCTs of metformin versus placebo for outpatient treatment of SARS‐CoV‐2 infection. In this TTE, most prescriptions occurred within 2 days of the infection, suggesting successful identification of individuals who were seeking metformin or a control medication for their SARS‐CoV‐2 infection. Metformin prescription within the week following documented SARS‐CoV‐2 infection was associated with a 53% reduction in LC/D over 6 months in this primarily Omicron‐infected sample. These results are also consistent with a recent sequential target trial analysis in the United Kingdom that assessed metformin started within 90 days of infection, HR 0.36 (95% CI 0.32–0.41) [57].

In the COVID‐OUT trial, LC was ascertained by asking participants if a medical provider had diagnosed them with LC, and then obtaining medical records to confirm [15]. The overall incidence of LC in COVID‐OUT and this TTE is similar, 8.3% over 10 months and 6.3% over 6 months, respectively. These proportions are also similar to the 10% identified as having LC in the RECOVER prospective acute cohort [58].

The absolute risk reduction with metformin was similar in this TTE (4.4%) and the COVID‐OUT trial (4.1%). The absolute risk reduction in ACTIV‐6 was lower (−0.7%), but the relative risk reduction (RR 0.50, 95% CI 0.16 < 1.0) was similar to this TTE, the COVID‐OUT trial, and the UK target trial [59]. The lower absolute risk reduction reflects the lower incidence of clinician‐diagnosed Long COVID in ACTIV‐6, which is consistent with emerging data about general incidence [60, 61].

Unlike COVID‐OUT, which limited enrollment to those with obesity or overweight, this TTE included a sample that is more heavily weighted toward the Omicron era, and is less restrictive with respect to age, comorbidity, or BMI, although the number of patients with documented BMI under 25 kg/m^2^ in the metformin group was small. ACTIV‐6 also did not have a minimum BMI requirement, and neither ACTIV‐6 nor the UK target trial excluded individuals with a known prior SARS‐CoV‐2 infection [57, 59].

A smaller point estimate with earlier evidence of medication use was also similar between the current analysis and COVID‐OUT, in which participants who started metformin in < 4 days of symptom onset had a 63% lower likelihood of receiving a diagnosis of LC over the subsequent 10 months [15]. However, in both an RCT and this TTE, subgroups lack precision and should be interpreted with caution. A larger effect when therapy is initiated earlier in infection would align with studies that it is important to start antiviral against SARS‐CoV‐2 early to reduce viral replication [62, 63]. Metformin lowered the viral load in two RCTs, and in vitro studies found metformin lowered SARS‐CoV‐2 RNA while increasing cell viability [11, 12, 13, 16]. Two prospective cohorts also suggest lower incidence of LC in those with prevalent metformin use [64, 65].

Small number bias may influence the results in this TTE. We considered other approaches to defining the treatment window to increase the sample size, such as allowing the window to start on Day −1. However, we were concerned that individuals who received a prescription before Day 0 may have been receiving that prescription for another indication and thus would not be starting it while acutely ill with COVID‐19.

The risk ratio in the subgroup excluding those with a nirmatrelvir–ritonavir prescription was slightly higher, generating the hypothesis that those receiving nirmatrelvir–ritonavir prescription in addition to a new metformin prescription had a lower risk ratio than the sample as a whole.

### 4.1 Limitations

Observational analyses may be limited by missing data and misclassification of exposures, outcomes, and confounders. For example, the use of nirmatrelvir–ritonavir may not be captured in the EHR because individuals were able to obtain nirmatrelvir–ritonavir directly from pharmacies without seeing their physician. A significant limitation is the lack of vaccination data and residual unmeasured confounding. Proxy variables for vaccination status were not explored. However, the use of an active comparator should distribute unmeasured confounders more evenly between the metformin and comparator groups than comparing metformin starters to nonstarters. In observational data, we do not know when symptoms started nor if prescriptions were filled and taken, but it is important to assess clinical trial findings in real‐world settings. The UK target trial had similar results in their per‐protocol assessment of those who filled metformin prescriptions [57]. Because of home testing, the exact number and date of SARS‐CoV‐2 infections may be missing. There may have been undiagnosed diabetes or prediabetes in the metformin group given the elevated mean hemoglobin A1C in that group, but prevalent metformin use in the metformin group is less of a concern than prevalent metformin use in the comparator group. Several other factors could affect outcome ascertainment, such as vague symptoms, access to healthcare, and pre‐existing conditions, but challenges in defining and identifying LC are not unique to this analysis.

## 5 Conclusions

In a TTE, metformin prescribed within a week of SARS‐CoV‐2 infection resulted in a 53% lower risk of LC/D in a primarily Omicron‐infected population from a nationwide deidentified EHR database. Anything between an 11% and 75% reduction in risk is highly compatible with our data. This result is consistent with both randomized trials that were emulated and other emerging observational analyses. While observational data alone should be interpreted with caution because there may be missingness in exposure, outcome, and confounder variables, the replication of results in clinical trials is important for informing treatment guidelines. Observational data improve generalizability beyond individuals who are eligible for and consent for clinical trials.

## Author Contributions

Conceptualization: Carolyn T. Bramante, Til Stürmer, John B. Buse, Christopher Lindsell, Thomas Stewart, David Sahner, and Sarah E. Dunsmore. Data curation: Talia D. Wiggen, Steve Makkar, and Steven G. Johnson. Formal analysis: Talia D. Wiggen, Steven G. Johnson, and Jared D. Huling. Investigation: Carolyn T. Bramante, John B. Buse, Eric Topol, Andrew Toler, Taylor Estepp, and Russell L. Rothman. Methodology: Til Stürmer, Jared D. Huling, Christopher Lindsell, Thomas Stewart, Steve Makkar, and Steven G. Johnson. Resources: Carolyn T. Bramante and Sarah E. Dunsmore. Visualization: Talia D. Wiggen. Writing – original draft: Carolyn T. Bramante and Steven G. Johnson. Writing – review and editing: Til Stürmer, Jared D. Huling, John B. Buse, Christopher Lindsell, Thomas Stewart, Russell L. Rothman, David Sahner, Sarah E. Dunsmore, Eric Topol, Talia D. Wiggen, Steve Makkar, Andrew Toler, Taylor Estepp, and Steven G. Johnson.

## Disclosure

The N3C Publication Committee confirmed that this manuscript MSID:2076.771 is in accordance with N3C data use and attribution policies; however, this content is solely the responsibility of the authors and does not necessarily represent the official views of the National Institutes of Health or the N3C program. Authorship was determined using the ICMJE recommendations.

## Ethics Statement

The N3C data transfer to NCATS is performed under a Johns Hopkins University Reliance Protocol # IRB00249128 or individual site agreements with NIH. The N3C Data Enclave is managed under the authority of the NIH; information can be found at https://ncats.nih.gov/n3c/resources.

## Conflicts of Interest

Drs. Bramante, Dunsmore, Stewart, Rothman, and Lindsell were on the executive committee for the ACTIV‐6 trial. Dr. Bramante is the principal investigator of the COVID‐OUT trial.

## Supporting information


Supporting File

## Data Availability

The data in the N3C are freely available to anyone with an approved data use agreement. The code and codesets are available in GitHub, https://github.com/UMN-IHI/phastr-metformin.
