# Supplementary material for: Metformin at the Time of COVID‐19 Infection and Risk of Long COVID: A Target Trial Emulation Study
Source: J Med Virol. 2026 Oct 29;98(11):e71190. doi: 10.1002/jmv.71190 (PMC13717933; doi:10.1002/jmv.71190)
Supplement: Supplementary file 1 [file JMV-98-e71190-s001.docx]

**Supplementary Appendix.**

**Table Of Contents**

| **Description** | **Title** | **Page** |
| --- | --- | --- |
| Additional information about the N3C | Text Box 1 | 1 |
|  | | |
| **Additional information about the outcome** | | |
| Cumulative incidence of the outcome components | Table S1 | 2 |
|  | | |
| **Additional information about the primary analytic sample** | | |
| Cohort balance of the standardized mean difference | Figure S1 | 3 |
| Medications prescribed after index date | Table S2 | 3 |
| Comprehensive list of all demographic variables, before weighting | Table S3 | 11 |
| Comprehensive list of all demographic variables, after weighting | Table S4 | 13 |
| Rationale for criteria applied in Figure 1 to create the analytic cohorts. | Table S5 | 15 |
|  | | |
| **Cumulative Incidence Curves for Subgroups** | | |
| Infected after the start of the Omicron Era | Figure S2 | 4 |
| Infected before the Omicron Era | Figure S3 | 5 |
| Excluding those with new use of Paxlovid | Figure S4 | 6 |
|  | | |
| **Cumulative Incidence Curves for Sensitivity analyses** | | |
| Excluding Fluvoxamine | Figure S5 | 7 |
| Excluding Ivermectin | Figure S6 | 8 |
| Excluding Control Indications | Figure S7 | 9 |
| Unadjusted Analyses | Figure S8 | 10 |
|  | | |
| **Additional manuscript information** | | |
| Full N3C Acknowledgements | Text Box 2 | 15 |

**Text Box 1.**

The National COVID Cohort Collaborative (N3C) is a centralized repository of real-world, de-identified electronic health record data. The infrastructure for this is supported by the National Institutes of Health (NIH). The N3C includes detailed information on inpatient and outpatient clinical encounters including: procedures, diagnoses, ordered and administered medications, demographic data, vitals, and lab orders and results.

Records in N3C are aggregated from the participating clinical organizations in the United States, known as data partners, and then harmonized using the Observational Medical Outcomes Partnership (OMOP) data model. The data are subjected to quality review and checks.

This resource was possible because of the patients whose information is included within the data and the organizations (<https://ncats.nih.gov/n3c/resources/data-contribution/data-transfer-agreement-signatories>) and scientists who have contributed to the on-going development of this community resource

| **Supplemental Table 1**. Cumulative incidence of the outcome components.* | **Days 0 - 1** | | | **Days 0 - 6** | | | **Days 0 - 14** | | |
| --- | --- | --- | --- | --- | --- | --- | --- | --- | --- |
|  | Overall  (n= 6,858) | Metformin | Control | Overall  (n= 9,660) | Metformin  (n=248) | Control  (n=9,412) | Overall  (n=12,806) | Metformin  (n=438) | Control  (n=12,368) |
| **Unweighted frequencies** | | | | | | | | | |
| Long Covid/Death | 305 (4.4%) | < 2.5% | < 4.6% | 674 (7.0%) | 10 (4.0%) | 664 (7.1%) | 1103 (8.6%) | 33 (7.5%) | 1070 (8.7%) |
| Death | 61 (0.9%) | 0 | <1% | 160 (1.7%) | < 1.7% | < 1.8% | 245 (1.9%) | 12 (2.7%) | 233 (1.9%) |
| LC (U09.9 + Computable phenotype) | 251 (3.7%) | < 2.5% | < 3.8% | 529 (5.5%) | < 2.9% | < 5.6% | 883 (6.9%) | 22 (5%) | 861 (7%) |
| **Weighted frequencies** | | | | | | | | | |
| Long Covid/Death | 4.3% | < 2.5% | < 6.3% | 6.3% | 4% | 8.5% | 8.8% | 7.5% | 10% |
| Death | 0.8% | 0 | 1.6% | 2.2% | < 1.7% | < 2.8% | 2.7% | 2.7% | 2.6% |
| LC (U09.9 + Computable phenotype) | 3.5% | < 2.5% | < 4.7% | 4.4% | < 2.9% | < 6% | 6.3% | 5% | 7.5% |
| Abbreviations: LC=Long Covid  *Of note, individuals could have LC (by U09.9 or computable phenotype) and then die. | | | | | | | | | |

Table of Contents

**Supplemental Figure 1:** Cohort balance of the standardized mean differences (SMD) before weighting (in pink), and after weighting (in teal), for the primary analysis.

| **Supplemental Table 2. Medications prescribed after the index event** | | | |
| --- | --- | --- | --- |
|  | **Metformin**  **(n=248)** | **Control**  **(n=9,412)** | **SMD** |
| Fluticasone, after | 22 (0.09) | 8,435 (0.90) | 2.739 |
| Fluvoxamine, after | 0 (0.00) | 105 (0.01) | 0.15 |
| Montelukast, after | < 10 (0.03) | 880 (0.09) | 0.276 |
| Ivermectin, after | 0 (0.00) | 318 (0.03) | 0.264 |
| Control Meds, all, after | 25 (0.10) | 9,412 (1.00) | 4.224 |
| Metformin, after | 248 (1.00) | 14 (0.00) | 36.641 |
| **Other Covid Treatments received 0 to 7 days relative to infection** | | | |
| Dexamethasone, after | 45 (0.18) | 863 (0.09) | 0.264 |
| Molnupiravir, after | < 10 (0.01) | 113 (0.01) | 0.001 |
| Ritonavir, after | < 10 (0.02) | 98 (0.01) | 0.05 |
| Nirmatrelvir, after | < 10 (0.02) | 129 (0.01) | 0.02 |
| Paxlovid, after | < 10 (0.02) | 354 (0.04) | 0.133 |
| Remdesivir, after | 49 (0.20) | 709 (0.08) | 0.362 |
| Control Days from Index, mean (SD) | 3.04 (3.79) | 1.21 (1.87) | 0.612 |
| Metformin Days from Index, mean (SD) | 2.09 (2.14) | 9.36 (2.71) | 2.978 |

Table of Contents

**Supplemental Figure 2**

Cumulative incidence curves for the outcome of Long Covid/Death in the 180 days after a metformin prescription (blue) or control prescription (red). The left panel is those receiving a prescription on days 0 to 6 from infection; the right panel is those receiving a prescription on days 0 to 14 from infection. The increase in outcomes at day 90 reflects the computable phenotype, which cannot be computed until 90 days after infection. Per data use requirements in the National Covid Cohort Collaborative (N3C), the number at risk is not shown for the starting cohort sizes for the 0 to 6 and 0 to 14, due the ability to calculate small cell sizes.

Table of Contents

**Supplemental Figure 3**

Cumulative incidence curves for the outcome of Long Covid/Death in the 180 days after a metformin prescription (blue) or control prescription (red). The left panel is those receiving a prescription on days 0 to 6 from infection; the right panel is those receiving a prescription on days 0 to 14 from infection. The increase in outcomes at day 90 reflects the computable phenotype, which cannot be computed until 90 days after infection. Per data use requirements in the National Covid Cohort Collaborative (N3C), the number at risk is not shown for the starting cohort sizes for the 0 to 6 and 0 to 14, due the ability to calculate small cell sizes.

Table of Contents

We note that the number with new Paxlovid use was too small to analyze, we could only analyze those remaining after excluding those with new Paxlovid use.

**Supplemental Figure 4**

Cumulative incidence curves for the outcome of Long Covid/Death in the 180 days after a metformin prescription (blue) or control prescription (red). The left panel is those receiving a prescription on days 0 to 6 from infection; the right panel is those receiving a prescription on days 0 to 14 from infection. The increase in outcomes at day 90 reflects the computable phenotype, which cannot be computed until 90 days after infection. Per data use requirements in the National Covid Cohort Collaborative (N3C), the number at risk is not shown for the starting cohort sizes for the 0 to 6 and 0 to 14, due the ability to calculate small cell sizes.

Table of Contents

**Supplemental Figure 5**

Cumulative incidence curves for the outcome of Long Covid/Death in the 180 days after a metformin prescription (blue) or control

prescription (red). The left panel is those receiving a prescription on days 0 to 6 from infection; the right panel is those receiving a prescription on

days 0 to 14 from infection. The increase in outcomes at day 90 reflects the computable phenotype, which cannot be computed until 90 days after

infection. Per data use requirements in the National Covid Cohort Collaborative (N3C), the number at risk is not shown for the starting cohort sizes

for the 0 to 6 and 0 to 14, due the ability to calculate small cell sizes.

Table of Contents

**Supplemental Figure 6**

Cumulative incidence curves for the outcome of Long Covid/Death in the 180 days after a metformin prescription (blue) or control prescription (red). The left panel is those receiving a prescription on days 0 to 6 from infection; the right panel is those receiving a prescription on days 0 to 14 from infection. The increase in outcomes at day 90 reflects the computable phenotype, which cannot be computed until 90 days after infection. Per data use requirements in the National Covid Cohort Collaborative (N3C), the number at risk is not shown for the starting cohort sizes for the 0 to 6 and 0 to 14, due the ability to calculate small cell sizes.

Table of Contents

**Supplemental Figure 7**

Cumulative incidence curves for the outcome of Long Covid/Death in the 180 days after a metformin prescription (blue) or control prescription (red). The left panel is those receiving a prescription on days 0 to 6 from infection; the right panel is those receiving a prescription on days 0 to 14 from infection. The increase in outcomes at day 90 reflects the computable phenotype, which cannot be computed until 90 days after infection. Per data use requirements in the National Covid Cohort Collaborative (N3C), the number at risk is not shown for the starting cohort sizes for the 0 to 6 and 0 to 14, due the ability to calculate small cell sizes.

Table of Contents

**Supplemental Figure 8**

Cumulative incidence curves for the outcome of Long Covid/Death in the 180 days after a metformin prescription (blue) or control prescription (red). The left panel is those receiving a prescription on days 0 to 6 from infection; the right panel is those receiving a prescription on days 0 to 14 from infection. The increase in outcomes at day 90 reflects the computable phenotype, which cannot be computed until 90 days after infection. Per data use requirements in the National Covid Cohort Collaborative (N3C), the number at risk is not shown for the starting cohort sizes for the 0 to 6 and 0 to 14, due the ability to calculate small cell sizes.

Table of Contents

| **Table S3. Comprehensive list of all variables included in balancing, before weighting.** | **Metformin**  **(n=248)** | **Control**  **(n=9,412)** | **SMD** |
| --- | --- | --- | --- |
| **Demographics** |  |  |  |
| Age, mean (SD) | 53.31 (16.48) | 45.28 (20.66) | 0.43 |
| **Age Category** |  |  |  |
| 0 to 21 | < 10 (0.04) | <1,290 (<0.15) | 0.383 |
| 21 to 45 | < 70 (<0.28) | 3,454 (0.37) | 0.2 |
| 46 to 65 | 116 (0.47) | 2,964 (0.31) | 0.317 |
| 66 plus | 56 (0.23) | 1,702 (0.18) | 0.112 |
| Missing | 0 (0.00) | < 10 (0.00) | 0.029 |
| Female | 113 (0.46) | 6,310 (0.67) | 0.444 |
| **Race, Ethnicity** |  |  |  |
| American Indian or Alaska Native Non-Hispanic | < 10 (0.00) | 42 (0.00) | 0.007 |
| Asian Non-Hispanic | < 10 (0.02) | 236 (0.03) | 0.063 |
| Black or African American Non-Hispanic | 40 (0.16) | 1,638 (0.17) | 0.034 |
| Hispanic or Latino Any Race | 39 (0.16) | 1,251 (0.13) | 0.069 |
| Native Hawaiian or Other Pacific Islander Non-Hispanic | 0 (0.00) | 24 (0.00) | 0.072 |
| White Non-Hispanic | 153 (0.62) | 5,807 (0.62) | 0 |
| Office Visits, 0-6 months before | 6.28 (9.56) | 7.87 (9.89) | 0.163 |
| Office Visits, 6-12 months before | 5.19 (7.95) | 7.34 (9.17) | 0.251 |
| Office Visits, after | 18.74 (22.46) | 16.10 (19.89) | 0.125 |
| BMI, mean (SD) | 35.91 (9.30) | 29.64 (8.26) | 0.712 |
| **BMI Category** |  |  |  |
| 0 to 18.5 | < 10 (0.00) | 351 (0.04) | 0.235 |
| 18.5 to 24.9 | < 10 (0.03) | 1,651 (0.18) | 0.483 |
| 25.0 to 29.9 | 28 (0.11) | 1,849 (0.20) | 0.233 |
| 30.0 plus | 94 (0.38) | 2,907 (0.31) | 0.148 |
| Missing | 117 (0.47) | 2,654 (0.28) | 0.399 |
| **Medications prior to index event**  ACEi | 21 (0.08) | 592 (0.06) | 0.083 |
| ARB | 18 (0.07) | 552 (0.06) | 0.056 |
| Statins | 18 (0.07) | 1,065 (0.11) | 0.14 |
| Anticoagulant | 14 (0.06) | 492 (0.05) | 0.018 |
| Aspirin | 11 (0.04) | 414 (0.04) | 0.002 |
| Steroids | 61 (0.25) | 2,776 (0.29) | 0.11 |
| SSRIs | 18 (0.07) | 1,197 (0.13) | 0.183 |
| DPP4i | < 10 (0.00) | < 10 (0.00) | 0.086 |
| GLP-1 RA | < 10 (0.02) | 44 (0.00) | 0.14 |
| SGLT-2 inhibitor | < 10 (0.00) | 12 (0.00) | 0.054 |
| Sulfonylureas | < 10 (0.01) | < 10 (0.00) | 0.148 |
| Thiazolidinediones | 0 (0.00) | 0 (0.00) | 0 |
| Outpatient Insulin | 14 (0.06) | 73 (0.01) | 0.279 |
| Other Diabetes meds | 0 (0.00) | < 10 (0.00) | 0.021 |
| **Clinical conditions prior to index event**  CAD | 15 (0.06) | 564 (0.06) | 0.002 |
| Cancer | 12 (0.05) | 660 (0.07) | 0.092 |
| CKD, Stage 1-3 | < 10 (0.02) | 341 (0.04) | 0.126 |
| Heart Failure | 10 (0.04) | 342 (0.04) | 0.021 |
| Hypertension | 71 (0.29) | 2,850 (0.30) | 0.036 |
| Liver Disease | < 10 (0.01) | 123 (0.01) | 0.049 |
| NAFLD | 10 (0.04) | 315 (0.03) | 0.036 |
| Adjustment d/o | < 10 (0.00) | 121 (0.01) | 0.097 |
| Dysthymia | < 10 (0.01) | 116 (0.01) | 0.002 |
| SAD | < 10 (0.02) | 143 (0.02) | 0.038 |
| Hypomania | < 10 (0.02) | 186 (0.02) | 0.027 |
| Dysphoria | 0 (0.00) | 14 (0.00) | 0.055 |
| Insomnia | < 10 (0.03) | 308 (0.03) | 0.003 |
| Bipolar Disease | < 10 (0.02) | 249 (0.03) | 0.014 |
| Mood Disorder | < 10 (0.02) | 162 (0.02) | 0.022 |
| Binge eating d/o | < 10 (0.00) | 43 (0.00) | 0.008 |
| Bulimia | 0 (0.00) | < 10 (0.00) | 0.033 |
| GAD | 13 (0.05) | 926 (0.10) | 0.175 |
| Depression | 21 (0.08) | 1,295 (0.14) | 0.169 |
| OCD | < 10 (0.00) | 64 (0.01) | 0.038 |
| Panic d/o | < 10 (0.01) | 309 (0.03) | 0.14 |
| Post Traumatic Stress d/o | < 10 (0.03) | 258 (0.03) | 0.005 |
| Social Anxiety d/o | < 10 (0.01) | 39 (0.00) | 0.05 |
| Asthma | 23 (0.09) | 1,970 (0.21) | 0.33 |
| COPD | < 10 (0.04) | 511 (0.05) | 0.087 |

| Bronchiolitis obliterans | 0 (0.00) | 23 (0.00) | 0.07 |
| --- | --- | --- | --- |
| Eosinophilic esophagitis | 0 (0.00) | 31 (0.00) | 0.081 |
| Post-hematopoietic cell transplantation | 0 (0.00) | < 10 (0.00) | 0.025 |
| Exercised-induced Asthma | 0 (0.00) | 66 (0.01) | 0.119 |
| Exercised-induced bronchoconstriction | 0 (0.00) | 66 (0.01) | 0.119 |
| Allergic rhinitis | 10 (0.04) | 1,202 (0.13) | 0.319 |
| Prediabetes | 0 (0.00) | 0 (0.00) | 0 |
| Type 1 DM | 0 (0.00) | 0 (0.00) | 0 |
| Type 2 DM | 0 (0.00) | 0 (0.00) | 0 |
| Gestational DM | 0 (0.00) | 0 (0.00) | 0 |
| Antipsychotic weight gain | 0 (0.00) | 0 (0.00) | 0 |
| Polycystic Ovarian Syndrome | 0 (0.00) | 0 (0.00) | 0 |
| **Laboratory values**  HbA1c>=6.5 | 35 (0.14) | 68 (0.01) | 0.529 |
| Last HbA1c, mean (SD) | 7.09 (2.39) | 5.39 (0.52) | 0.983 |
| Creatinine, mean (SD) | 0.92 (0.57) | 0.93 (0.72) | 0.011 |
| estimated GFR, mean (SD) | 97.36 (18.78) | 101.53 (24.16) | 0.193 |
| **COVID Variant Epoch**  Ancestral | 21 (0.08) | 391 (0.04) | 0.178 |
| Alpha | 70 (0.28) | 1,651 (0.18) | 0.256 |
| Delta | 34 (0.14) | 1,691 (0.18) | 0.117 |
| Omicron | 123 (0.50) | 5,664 (0.60) | 0.214 |
| **Control Days from Index Category** |  |  |  |
| -2 to 0 | < 10 (0.04) | 5,751 (0.61) | 1.557 |
| 1 to 3 | < 10 (0.03) | 2,165 (0.23) | 0.613 |
| 3 to 5 | < 10 (0.01) | 1,018 (0.11) | 0.438 |
| 5 to 7 | < 10 (0.02) | 478 (0.05) | 0.194 |
| 10 to 14 | < 10 (0.01) | 0 (0.00) | 0.128 |
| 14 plus | 223 (0.90) | 0 (0.00) | 4.224 |
| **Metformin Days from Index Category**  - 2 to 0 | 94 (0.38) | 0 (0.00) | 1.105 |
| 1 to 3 | 85 (0.34) | 0 (0.00) | 1.021 |
| 3 to 5 | 43 (0.17) | < 10 (0.00) | 0.647 |
| 5 to 7 | 26 (0.10) | < 10 (0.00) | 0.481 |
| 7 to 10 | 0 (0.00) | < 10 (0.00) | 0.025 |
| 10 to 14 | 0 (0.00) | < 10 (0.00) | 0.036 |
| 14 plus | 0 (0.00) | 9,398 (1.00) | 36.641 |

Variables are based on ICD code (co-morbidities); prescriptions; dates; vitals, and lab values as appropriate.

Table of Contents

| **Table S4. Comprehensive list of all variables included in balancing, after weighting.** | **Metformin**  **(n=248)** | **Control**  **(n=248.1)** | **SMD** |
| --- | --- | --- | --- |
| **Demographics** |  |  |  |
| Age, mean (SD) | 53.31 (16.45) | 52.74 (17.25) | 0.034 |
| **Age Category** |  |  |  |
| 0 to 21 | < 10 (0.03) | < 10 (0.03) | 0.000 |
| 21 to 45 | 68 (0.27) | 68.0 (0.27) | 0.000 |
| 46 to 65 | 116 (0.47) | 116.0 (0.47) | 0.000 |
| 66 plus | 56 (0.23) | 56.0 (0.23) | 0.000 |
| Missing | 0 (0.00) | < 10 (0.00) | 0.004 |
| Female | 113 (0.46) | 113.1 (0.46) | 0.000 |
| **Race, Ethnicity** |  |  |  |
| American Indian or Alaska Native Non-Hispanic | < 10 (0.00) | < 10 (0.00) | 0.000 |
| Asian Non-Hispanic | < 10 (0.02) | < 10 (0.02) | 0.000 |
| Black or African American Non-Hispanic | 40 (0.16) | 40.0 (0.16) | 0.000 |
| Hispanic or Latino Any Race | 39 (0.16) | 39.0 (0.16) | 0.000 |
| Native Hawaiian or Other Pacific Islander Non-Hispanic | 0 (0.00) | < 10 (0.00) | 0.009 |
| White Non-Hispanic | 153 (0.62) | 153.1 (0.62) | 0.000 |
| Office Visits, 0-6 months before | 6.28 (9.54) | 7.38 (10.94) | 0.107 |
| Office Visits, 6-12 months before | 5.19 (7.94) | 6.38 (8.52) | 0.144 |
| Office Visits, after | 18.74 (22.42) | 18.21 (21.76) | 0.024 |
| BMI, mean (SD) | 35.91 (9.27) | 35.90 (11.05) | 0.000 |
| BMI Category |  |  |  |
| 0 to 18.5 | < 10 (0.00) | < 10 (0.00) | 0.007 |
| 18.5 to 24.9 | < 10 (0.03) | 14.1 (0.06) | 0.120 |
| 25.0 to 29.9 | 28 (0.11) | 28.5 (0.12) | 0.007 |
| 30.0 plus | 94 (0.38) | 87.5 (0.35) | 0.055 |
| Missing | 117 (0.47) | 117.0 (0.47) | 0.000 |
| **Medications prior to index event**  ACEi | 21 (0.08) | 21.0 (0.08) | 0.000 |
| ARB | 18 (0.07) | 18.0 (0.07) | 0.000 |
| Statins | 18 (0.07) | 18.0 (0.07) | 0.000 |
| Anticoagulant | 14 (0.06) | 14.0 (0.06) | 0.000 |
| Aspirin | 11 (0.04) | 11.0 (0.04) | 0.000 |
| Steroids | 61 (0.25) | 61.0 (0.25) | 0.000 |
| SSRIs | 18 (0.07) | 18.0 (0.07) | 0.000 |
| DPP4i | < 10 (0.00) | < 10 (0.00) | 0.000 |
| GLP-1 RA | < 10 (0.02) | < 10 (0.02) | 0.000 |
| SGLT-2 inhibitor | < 10 (0.00) | < 10 (0.00) | 0.001 |
| Sulfonylureas | < 10 (0.01) | < 10 (0.01) | 0.000 |
| Thiazolidinediones | 0 (0.00) | 0.0 (0.00) | 0.000 |
| Outpatient Insulin | 14 (0.06) | 14.0 (0.06) | 0.000 |
| Other Diabetes meds | 0 (0.00) | < 10 (0.00) | 0.002 |
| **Clinical conditions prior to index event**  CAD | 15 (0.06) | 15.0 (0.06) | 0.000 |
| Cancer | 12 (0.05) | 12.0 (0.05) | 0.000 |
| CKD, Stage 1-3 | < 10 (0.02) | < 10 (0.02) | 0.000 |
| Heart Failure | 10 (0.04) | 10.0 (0.04) | 0.000 |
| Hypertension | 71 (0.29) | 71.0 (0.29) | 0.000 |
| Liver Disease | < 10 (0.01) | < 10 (0.01) | 0.000 |
| NAFLD | 10 (0.04) | 10.0 (0.04) | 0.000 |
| Adjustment d/o | < 10 (0.00) | < 10 (0.00) | 0.000 |
| Dysthymia | < 10 (0.01) | < 10 (0.01) | 0.000 |
| SAD | < 10 (0.02) | < 10 (0.02) | 0.000 |
| Hypomania | < 10 (0.02) | < 10 (0.02) | 0.000 |
| Dysphoria | 0.0 (0.00) | < 10 (0.00) | 0.007 |
| Insomnia | < 10 (0.03) | < 10 (0.03) | 0.000 |
| Bipolar Disease | < 10 (0.02) | < 10 (0.02) | 0.000 |
| Mood Disorder | < 10 (0.02) | < 10 (0.02) | 0.000 |
| Binge eating d/o | < 10 (0.00) | < 10 (0.00) | 0.000 |
| Bulimia | 0 (0.00) | < 10 (0.00) | 0.005 |
| GAD | 13 (0.05) | 13.0 (0.05) | 0.000 |
| Depression | 21 (0.08) | 21.0 (0.08) | 0.000 |
| OCD | < 10 (0.00) | < 10 (0.00) | 0.000 |
| Panic d/o | < 10 (0.01) | < 10 (0.01) | 0.000 |
| Post Traumatic Stress d/o | < 10 (0.03) | < 10 (0.03) | 0.000 |
| Social Anxiety d/o | < 10 (0.01) | < 10 (0.01) | 0.000 |
| Asthma | 23 (0.09) | 23.0 (0.09) | 0.000 |
| COPD | < 10 (0.04) | < 10 (0.04) | 0.000 |
| Bronchiolitis obliterans | 0 (0.00) | < 10 (0.00) | 0.010 |
| Eosinophilic esophagitis | 0 (0.00) | < 10 (0.00) | 0.010 |
| Post-hematopoietic cell transplantation | 0 (0.00) | < 10 (0.00) | 0.003 |
| Exercised-induced Asthma | 0 (0.00) | < 10 (0.00) | 0.015 |
| Exercised-induced bronchoconstriction | 0 (0.00) | < 10 (0.00) | 0.015 |
| Allergic rhinitis | 10 (0.04) | 10.0 (0.04) | 0.000 |
| Prediabetes | 0 (0.00) | 0.0 (0.00) | 0.000 |
| Type 1 DM | 0 (0.00) | 0.0 (0.00) | 0.000 |
| Type 2 DM | 0 (0.00) | 0.0 (0.00) | 0.000 |
| Gestational DM | 0 (0.00) | 0.0 (0.00) | 0.000 |
| Antipsychotic weight gain | 0 (0.00) | 0.0 (0.00) | 0.000 |
| Polycystic Ovarian Syndrome | 0 (0.00) | 0.0 (0.00) | 0.000 |
| **Laboratory values**  HbA1c>=6.5 | 35.0 (0.14) | 35.0 (0.14) | 0.000 |
| Last HbA1c, mean (SD) | 7.09 (2.37) | 7.09 (2.76) | 0.000 |
| Creatinine, mean (SD) | 0.92 (0.57) | 0.92 (0.41) | 0.000 |
| estimated GFR, mean (SD) | 97.36 (18.74) | 97.36 (20.03) | 0.000 |
| **COVID Variant Epoch COVID Era**  Ancestral | 21 (0.08) | 20.9 (0.08) | 0.001 |
| Alpha | 70 (0.28) | 48.7 (0.20) | 0.203 |
| Delta | 34 (0.14) | 48.9 (0.20) | 0.162 |
| Omicron | 123 (0.50) | 129.4 (0.52) | 0.051 |
| **Control Days from Index Category** |  |  |  |
| -2 to 0 | < 10 (0.04) | 126.7 (0.51) | 1.257 |
| 1 to 3 | < 10 (0.03) | 66.9 (0.27) | 0.703 |
| 3 to 5 | < 10 (0.01) | 34.8 (0.14) | 0.521 |
| 5 to 7 | < 10 (0.02) | 19.6 (0.08) | 0.299 |
| 10 to 14 | < 10 (0.01) | 0.0 (0.00) | 0.128 |
| 14 plus | 223 (0.90) | 0.0 (0.00) | 4.224 |
| **Metformin Days from Index Category**  -2 to 0 | 94 (0.38) | 0.0 (0.00) | 1.105 |
| 1 to 3 | 85 (0.34) | 0.0 (0.00) | 1.021 |
| 3 to 5 | 43 (0.17) | < 10 (0.00) | 0.625 |
| 5 to 7 | 26 (0.10) | < 10 (0.00) | 0.475 |
| 7 to 10 | 0 (0.00) | < 10 (0.01) | 0.106 |
| 10 to 14 | 0 (0.00) | < 10 (0.00) | 0.051 |
| 14 plus | 0 (0.00) | 245.1 (0.99) | 12.764 |
| Variables are based on ICD code (co-morbidities); prescriptions; dates; vitals, and lab values as appropriate. | | | |

Table of Contents

| **Table S5. Rationale for exclusion criteria applied in Figure 1 to create the analytic cohorts.** | |
| --- | --- |
| **Exclusion Criterion** | **Rationale** |
| Does not have clinic visit in the 0 to 6 and 6 to 12 months prior to infection | Requiring prior system use improves the likelihood that cohort-defining prescriptions and confounders will be recorded. |
| Metformin prescription in the past 12 months | Current metformin users would not be eligible for a clinical trial that randomizes people to metformin. |
| Comparator prescription in the past 12 months | Current comparator users would not be eligible for a clinical trial that randomizes people to the comparator. |
| Hospitalized within -3 to 1 days of infection. | These individuals would not be eligible for one of the emulated clinical trials. |
| Contraindication for metformin (chronic kidney disease) | These individuals would not be eligible for a randomized clinical trial of metformin. |
| Indication for chronic metformin use | It is important to minimize metformin use in the comparator cohort in order to emulate the placebo condition of a randomized trial. |
| Sites that have never reported a U09.9 diagnosis code for LC. | Concern for misclassification of the outcome from these data partners. |
| LC or death before prescription. | These individuals would not be eligible for the emulated clinical trials. |

Text Box 2

Full N3C Acknowledgements

Individual Acknowledgements For Core Contributors

We gratefully acknowledge the following core contributors to N3C:

Adam B. Wilcox, Adam M. Lee, Alexis Graves, Alfred (Jerrod) Anzalone, Amin Manna, Amit Saha, Amy Olex, Andrea Zhou, Andrew E. Williams, Andrew Southerland, Andrew T. Girvin, Anita Walden, Anjali A. Sharathkumar, Benjamin Amor, Benjamin Bates, Brian Hendricks, Brijesh Patel, Caleb Alexander, Carolyn Bramante, Cavin Ward-Caviness, Charisse Madlock-Brown, Christine Suver, Christopher Chute, Christopher Dillon, Chunlei Wu, Clare Schmitt, Cliff Takemoto, Dan Housman, Davera Gabriel, David A. Eichmann, Diego Mazzotti, Don Brown, Eilis Boudreau, Elaine Hill, Elizabeth Zampino, Emily Carlson Marti, Emily R. Pfaff, Evan French, Farrukh M Koraishy, Federico Mariona, Fred Prior, George Sokos, Greg Martin, Harold Lehmann, Heidi Spratt, Hemalkumar Mehta, Hongfang Liu, Hythem Sidky, J.W. Awori Hayanga, Jami Pincavitch, Jaylyn Clark, Jeremy Richard Harper, Jessica Islam, Jin Ge, Joel Gagnier, Joel H. Saltz, Joel Saltz, Johanna Loomba, John Buse, Jomol Mathew, Joni L. Rutter, Julie A. McMurry, Justin Guinney, Justin Starren, Karen Crowley, Katie Rebecca Bradwell, Kellie M. Walters, Ken Wilkins, Kenneth R. Gersing, Kenrick Dwain Cato, Kimberly Murray, Kristin Kostka, Lavance Northington, Lee Allan Pyles, Leonie Misquitta, Lesley Cottrell, Lili Portilla, Mariam Deacy, Mark M. Bissell, Marshall Clark, Mary Emmett, Mary Morrison Saltz, Matvey B. Palchuk, Melissa A. Haendel, Meredith Adams, Meredith Temple-O'Connor, Michael G. Kurilla, Michele Morris, Nabeel Qureshi, Nasia Safdar, Nicole Garbarini, Noha Sharafeldin, Ofer Sadan, Patricia A. Francis, Penny Wung Burgoon, Peter Robinson, Philip R.O. Payne, Rafael Fuentes, Randeep Jawa, Rebecca Erwin-Cohen, Rena Patel, Richard A. Moffitt, Richard L. Zhu, Rishi Kamaleswaran, Robert Hurley, Robert T. Miller, Saiju Pyarajan, Sam G. Michael, Samuel Bozzette, Sandeep Mallipattu, Satyanarayana Vedula, Scott Chapman, Shawn T. O'Neil, Soko Setoguchi, Stephanie S. Hong, Steve Johnson, Tellen D. Bennett, Tiffany Callahan, Umit Topaloglu, Usman Sheikh, Valery Gordon, Vignesh Subbian, Warren A. Kibbe, Wenndy Hernandez, Will Beasley, Will Cooper, William Hillegass, Xiaohan Tanner Zhang. Details of contributions available at covid.cd2h.org/core-contributors

Data Partners with Released Data

The following institutions whose data is released or pending:

Available: Advocate Health Care Network — UL1TR002389: The Institute for Translational Medicine (ITM) • Aurora Health Care Inc — UL1TR002373: Wisconsin Network For Health Research • Boston University Medical Campus — UL1TR001430: Boston University Clinical and Translational Science Institute • Brown University — U54GM115677: Advance Clinical Translational Research (Advance-CTR) • Carilion Clinic — UL1TR003015: iTHRIV Integrated Translational health Research Institute of Virginia • Case Western Reserve University — UL1TR002548: The Clinical & Translational Science Collaborative of Cleveland (CTSC) • Charleston Area Medical Center — U54GM104942: West Virginia Clinical and Translational Science Institute (WVCTSI) • Children’s Hospital Colorado — UL1TR002535: Colorado Clinical and Translational Sciences Institute • Columbia University Irving Medical Center — UL1TR001873: Irving Institute for Clinical and Translational Research • Dartmouth College — None (Voluntary) Duke University — UL1TR002553: Duke Clinical and Translational Science Institute • George Washington Children’s Research Institute — UL1TR001876: Clinical and Translational Science Institute at Children’s National (CTSA-CN) • George Washington University — UL1TR001876: Clinical and Translational Science Institute at Children’s National (CTSA-CN) • Harvard Medical School — UL1TR002541: Harvard Catalyst • Indiana University School of Medicine — UL1TR002529: Indiana Clinical and Translational Science Institute • Johns Hopkins University — UL1TR003098: Johns Hopkins Institute for Clinical and Translational Research • Louisiana Public Health Institute — None (Voluntary) • Loyola Medicine — Loyola University Medical Center • Loyola University Medical Center — UL1TR002389: The Institute for Translational Medicine (ITM) • Maine Medical Center — U54GM115516: Northern New England Clinical & Translational Research (NNE-CTR) Network • Mary Hitchcock Memorial Hospital & Dartmouth Hitchcock Clinic — None (Voluntary) • Massachusetts General Brigham — UL1TR002541: Harvard Catalyst • Mayo Clinic Rochester — UL1TR002377: Mayo Clinic Center for Clinical and Translational Science (CCaTS) • Medical University of South Carolina — UL1TR001450: South Carolina Clinical & Translational Research Institute (SCTR) • MITRE Corporation — None (Voluntary) • Montefiore Medical Center — UL1TR002556: Institute for Clinical and Translational Research at Einstein and Montefiore • Nemours — U54GM104941: Delaware CTR ACCEL Program • NorthShore University HealthSystem — UL1TR002389: The Institute for Translational Medicine (ITM) • Northwestern University at Chicago — UL1TR001422: Northwestern University Clinical and Translational Science Institute (NUCATS) • OCHIN — INV-018455: Bill and Melinda Gates Foundation grant to Sage Bionetworks • Oregon Health & Science University — UL1TR002369: Oregon Clinical and Translational Research Institute • Penn State Health Milton S. Hershey Medical Center — UL1TR002014: Penn State Clinical and Translational Science Institute • Rush University Medical Center — UL1TR002389: The Institute for Translational Medicine (ITM) • Rutgers, The State University of New Jersey — UL1TR003017: New Jersey Alliance for Clinical and Translational Science • Stony Brook University — U24TR002306 • The Alliance at the University of Puerto Rico, Medical Sciences Campus — U54GM133807: Hispanic Alliance for Clinical and Translational Research (The Alliance) • The Ohio State University — UL1TR002733: Center for Clinical and Translational Science • The State University of New York at Buffalo — UL1TR001412: Clinical and Translational Science Institute • The University of Chicago — UL1TR002389: The Institute for Translational Medicine (ITM) • The University of Iowa — UL1TR002537: Institute for Clinical and Translational Science • The University of Miami Leonard M. Miller School of Medicine — UL1TR002736: University of Miami Clinical and Translational Science Institute • The University of Michigan at Ann Arbor — UL1TR002240: Michigan Institute for Clinical and Health Research • The University of Texas Health Science Center at Houston — UL1TR003167: Center for Clinical and Translational Sciences (CCTS) • The University of Texas Medical Branch at Galveston — UL1TR001439: The Institute for Translational Sciences • The University of Utah — UL1TR002538: Uhealth Center for Clinical and Translational Science • Tufts Medical Center — UL1TR002544: Tufts Clinical and Translational Science Institute • Tulane University — UL1TR003096: Center for Clinical and Translational Science • The Queens Medical Center — None (Voluntary) • University Medical Center New Orleans — U54GM104940: Louisiana Clinical and Translational Science (LA CaTS) Center • University of Alabama at Birmingham — UL1TR003096: Center for Clinical and Translational Science • University of Arkansas for Medical Sciences — UL1TR003107: UAMS Translational Research Institute • University of Cincinnati — UL1TR001425: Center for Clinical and Translational Science and Training • University of Colorado Denver, Anschutz Medical Campus — UL1TR002535: Colorado Clinical and Translational Sciences Institute • University of Illinois at Chicago — UL1TR002003: UIC Center for Clinical and Translational Science • University of Kansas Medical Center — UL1TR002366: Frontiers: University of Kansas Clinical and Translational Science Institute • University of Kentucky — UL1TR001998: UK Center for Clinical and Translational Science • University of Massachusetts Medical School Worcester — UL1TR001453: The UMass Center for Clinical and Translational Science (UMCCTS) • University Medical Center of Southern Nevada — None (voluntary) • University of Minnesota — UL1TR002494: Clinical and Translational Science Institute • University of Mississippi Medical Center — U54GM115428: Mississippi Center for Clinical and Translational Research (CCTR) • University of Nebraska Medical Center — U54GM115458: Great Plains IDeA-Clinical & Translational Research • University of North Carolina at Chapel Hill — UM1TR004406: North Carolina Translational and Clinical Science Institute • University of Oklahoma Health Sciences Center — U54GM104938: Oklahoma Clinical and Translational Science Institute (OCTSI) • University of Pittsburgh — UL1TR001857: The Clinical and Translational Science Institute (CTSI) • University of Pennsylvania — UL1TR001878: Institute for Translational Medicine and Therapeutics • University of Rochester — UL1TR002001: UR Clinical & Translational Science Institute • University of Southern California — UL1TR001855: The Southern California Clinical and Translational Science Institute (SC CTSI) • University of Vermont — U54GM115516: Northern New England Clinical & Translational Research (NNE-CTR) Network • University of Virginia — UL1TR003015: iTHRIV Integrated Translational health Research Institute of Virginia • University of Washington — UL1TR002319: Institute of Translational Health Sciences • University of Wisconsin-Madison — UL1TR002373: UW Institute for Clinical and Translational Research • Vanderbilt University Medical Center — UL1TR002243: Vanderbilt Institute for Clinical and Translational Research • Virginia Commonwealth University — UL1TR002649: C. Kenneth and Dianne Wright Center for Clinical and Translational Research • Wake Forest University Health Sciences — UL1TR001420: Wake Forest Clinical and Translational Science Institute • Washington University in St. Louis — UL1TR002345: Institute of Clinical and Translational Sciences • Weill Medical College of Cornell University — UL1TR002384: Weill Cornell Medicine Clinical and Translational Science Center • West Virginia University — U54GM104942: West Virginia Clinical and Translational Science Institute (WVCTSI)  Submitted: Icahn School of Medicine at Mount Sinai — UL1TR001433: ConduITS Institute for Translational Sciences • The University of Texas Health Science Center at Tyler — UL1TR003167: Center for Clinical and Translational Sciences (CCTS) • University of California, Davis — UL1TR001860: UCDavis Health Clinical and Translational Science Center • University of California, Irvine — UL1TR001414: The UC Irvine Institute for Clinical and Translational Science (ICTS) • University of California, Los Angeles — UL1TR001881: UCLA Clinical Translational Science Institute • University of California, San Diego — UL1TR001442: Altman Clinical and Translational Research Institute • University of California, San Francisco — UL1TR001872: UCSF Clinical and Translational Science Institute  NYU Langone Health Clinical Science Core, Data Resource Core, and PASC Biorepository Core — OTA-21-015A: Post-Acute Sequelae of SARS-CoV-2 Infection Initiative (RECOVER)  Pending: Arkansas Children’s Hospital — UL1TR003107: UAMS Translational Research Institute • Baylor College of Medicine — None (Voluntary) • Children’s Hospital of Philadelphia — UL1TR001878: Institute for Translational Medicine and Therapeutics • Cincinnati Children’s Hospital Medical Center — UL1TR001425: Center for Clinical and Translational Science and Training • Emory University — UL1TR002378: Georgia Clinical and Translational Science Alliance • HonorHealth — None (Voluntary) • Loyola University Chicago — UL1TR002389: The Institute for Translational Medicine (ITM) • Medical College of Wisconsin — UL1TR001436: Clinical and Translational Science Institute of Southeast Wisconsin • MedStar Health Research Institute — None (Voluntary) • Georgetown University — UL1TR001409: The Georgetown-Howard Universities Center for Clinical and Translational Science (GHUCCTS) • MetroHealth — None (Voluntary) • Montana State University — U54GM115371: American Indian/Alaska Native CTR • NYU Langone Medical Center — UL1TR001445: Langone Health’s Clinical and Translational Science Institute • Ochsner Medical Center — U54GM104940: Louisiana Clinical and Translational Science (LA CaTS) Center • Regenstrief Institute — UL1TR002529: Indiana Clinical and Translational Science Institute • Sanford Research — None (Voluntary) • Stanford University — UL1TR003142: Spectrum: The Stanford Center for Clinical and Translational Research and Education • The Rockefeller University — UL1TR001866: Center for Clinical and Translational Science • The Scripps Research Institute — UL1TR002550: Scripps Research Translational Institute • University of Florida — UL1TR001427: UF Clinical and Translational Science Institute • University of New Mexico Health Sciences Center — UL1TR001449: University of New Mexico Clinical and Translational Science Center • University of Texas Health Science Center at San Antonio — UL1TR002645: Institute for Integration of Medicine and Science • Yale New Haven Hospital — UL1TR001863: Yale Center for Clinical Investigation

Table of Contents
